# Sequencing by Binding rivals error-corrected Sequencing by Synthesis technology for accurate detection and quantification of minor (<0.1%) subpopulation variants

**DOI:** 10.21203/rs.3.rs-4391713/v1

**Published:** 2024-05-21

**Authors:** Christopher J. Allender, Candice Wike, Dean Ellis, Darrin Lemmer, Tanner Porter, Stephanie J.K. Pond, David M. Engelthaler

**Affiliations:** Translational Genomics Research Institute; Translational Genomics Research Institute; Translational Genomics Research Institute; Translational Genomics Research Institute; Translational Genomics Research Institute; Translational Genomics Research Institute; Translational Genomics Research Institute

**Keywords:** sequencing by synthesis (SBS), sequencing by binding (SBB), tuberculosis (TB), heteroresistance, single molecule overlapping reads (SMOR), ultra-rare mutation detection, NGS benchmarking

## Abstract

Detecting very minor (< 1%) subpopulations using next-generation sequencing is a critical need for multiple applications including detection of drug resistant pathogens and somatic variant detection in oncology. To enable these applications, wet lab enhancements and bioinformatic error correction methods have been developed for ‘sequencing by synthesis’ technology to reduce its inherent sequencing error rate. A recently available sequencing approach termed ‘sequencing by binding’ claims to have higher base calling accuracy data “out of the box.” This paper evaluates the utility of using ‘sequencing by binding’ for the detection of ultra-rare subpopulations down to 0.001%.

## Background

While next generation sequencing (NGS) has revolutionized genomics in oncology and infectious disease, challenges remain in characterizing cellular heterogeneity including the accurate measurement of minor populations (< 1%) due to the intrinsic error rates, particularly in ‘sequencing by synthesis’ (SBS) methods [1]. One critical use case for accurate low-frequency variant detection is the early identification of drug resistant subpopulations of *Mycobacterium tuberculosis* (*Mtb*), a phenomenon known as heteroresistance. Tuberculosis remains a leading global infectious disease problem due to many challenges associated with detecting and treating drug resistant *Mtb* infections. Sequencing-based analysis of resistance causing mutation loci in drug resistance-related *Mtb* genes has revealed both dominant and minor drug resistant subpopulations in patients exhibiting drug resistant infections [2–5]; accurate detection and quantification of these subpopulations can be significantly affected by sequencing error. A number of methods to physically and informatically reduce the effect of sequencing error of SBS to enable accurate analysis of low-level heteroresistant *Mtb* have been developed [6–9], but all add additional steps to library prep and often require cost-limited sequencing depth.

We have previously shown that single molecule overlapping reads (SMOR) analysis, which is a targeted NGS approach, reduces SBS sequencing error significantly and can be used to detect minor populations of drug resistant *Mtb* down to 0.1% of the total population [8–14]. Here, we assess the reliability of a novel ‘sequencing by binding’ (SBB) chemistry (Pacific Biosciences, PacBio) to sequence well defined *Mtb* drug resistance mutations in *katG* and *gyrA* genes (resulting in isoniazid and fluoroquinolone resistance, respectively) down to 0.01% without employing additional error correction methods (*e.g*., unique molecular identifiers (UMIs) or SMOR).

## Methods

In order assess the capabilities of targeted SBB sequencing to identify and quantify low to extremely low minor subpopulations, and compare to error-corrected and non-error-corrected targeted SBS sequencing, we created validated contrived mixtures, conducted PacBio SBB and Illumina SBS sequencing, and statistically compared sequencing outputs for sequencing accuracy and quantification precision. Methods in detail are as follows:

Contrived mixtures: Two plasmids were used as DNA template to create PCR products for making contrived drug resistant mutation mixtures at 10%, 1%, 0.1%, 0.01%, and 0.001%. A wildtype (WT) plasmid was created (Blue Heron Biotech, LLC) with *katG* and *gyrA* sequences that match the *Mycobacterium tuberculosis* H37Rv pan-susceptible strain. A second resistant (RS) plasmid was created (Blue Heron Biotech, LLC) with *katG* and *gyrA* mutations at *katG* g944c and *gyrA* a241g, which are mutations known to confer drug resistance in clinical isolates from tuberculosis patients [17]. Both plasmids were linearized with PciI restriction enzyme (New England Biolabs, Inc.) using manufacturer’s conditions and diluted to 10^3^ copies and used as DNA template for targeted sequencing PCR. This PCR contained primers (200 nM final conc.) with universal tails as previously described [9], Q5^®^ Hot Start High-Fidelity 2X Master Mix (New England Biolabs, Inc.) (1x final conc.), betaine (MilliporeSigma) (1M final conc.), and water to 30 μL. The cycling conditions were 98°C for 1 min; 35 cycles of 98°C for 15 sec, 60°C for 20 sec, and 72°C for 20sec; and 72°C for 5 min. The WT PCR products were diluted with water to 400 uL to make enough volume for subsequent 10-fold dilution series. Qubit dsDNA HS Assay Kit (Thermo Fisher Scientific, Inc.) quantification was used to measure, dilute, and confirm the RS PCR products were at the same concentration as the WT PCR products. With all PCR products at equimolar concentrations (i.e., for both *katG* and *gyrA*), three separate 10% mixtures were created by mixing 3 μL of RS PCR products into 27 μL of WT PCR products. Four additional 10-fold dilution series for each 10% mixture replicate were created using WT PCR products as the diluent. These universally-tailed PCR products (i.e., six different 10-fold dilution series) were purified with a 1.0x AMPure XP (Beckman Coulter, Inc) bead cleanup, eluted into 20 μl of water, processed further for either Illumina or PacBio SBB sequencing.

Illumina SBS sequencing: The purified PCR product mixtures were each barcoded with dual and unique 12 base pair indexing primers in a second PCR that also added adapters for sequencing on an Illumina DNA sequencer platform. This PCR contained 2 μL of the previous product, primers (400 nM final conc.) with barcodes and adapters as previously described [9], KAPA HiFi Hotstart ReadyMix (Roche Molecular Systems, Inc.) (1x final conc.), and water to 50 μL. The cycling conditions were 98°C for 2 min; 6 cycles of 98°C for 30 sec, 60°C for 20 sec, and 72°C for 30sec; and 72°C for 5 min. These PCR products were purified with a 0.8x AMPure XP (Beckman Coulter, Inc) bead cleanup and eluted into 40 μL of water. These individual libraries were quantified with a KAPA Library Quantification Low Rox Kit (Roche Molecular Systems, Inc.) on a QuantStudio^™^ 7 Flex Real-Time PCR System (Thermo Fisher Scientific, Inc.) and pooled in equimolar amounts before being combined with 20% PhiX sequencing control v3 (Illumina, Inc.), assessed with a High Sensitivity D5000 ScreenTape (Agilent Technologies, Inc.) on a 4200 TapeStation system (Agilent Technologies, Inc.), and finally sequenced on a MiSeq^®^ System (Illumina, Inc.) with a 600-cycle MiSeq Reagent Kit v3 (Illumina, Inc.).

PacBio SBB sequencing: The individual Illumina libraries were converted using the PacBio Onso Conversion protocol (PacBio 12-529-500). Briefly, a p5/p7 library (5–100 fmol) were added to PCR conversion primers (2.5ul), PCR master mix (2X) and Water up to 30ul. The cycling conditions were 98°C for 30 sec; 5 cycles of 98°C for 10 sec, 65°C for 30 sec, and 72°C for 30sec; and 72°C for 5 min. These PCR products were purified with a 1.6x AMPure XP (Beckman Coulter, Inc) bead cleanup and eluted into 52 μL of low TE. These individual libraries were quantified with a KAPA Library Quantification Low Rox Kit (Roche Molecular Systems, Inc.) on qPCR System and pooled in equimolar amounts before being combined with 10% Onso indexed library control (PacBio 102-529-900) assessed with a High Sensitivity D1000 ScreenTape (Agilent Technologies, Inc.) on a 4200 TapeStation system (Agilent Technologies, Inc.), and sequenced on a pre-commercial Onso instrument with a 300 cycle sequencing reagent kit.

**Figure 1:**
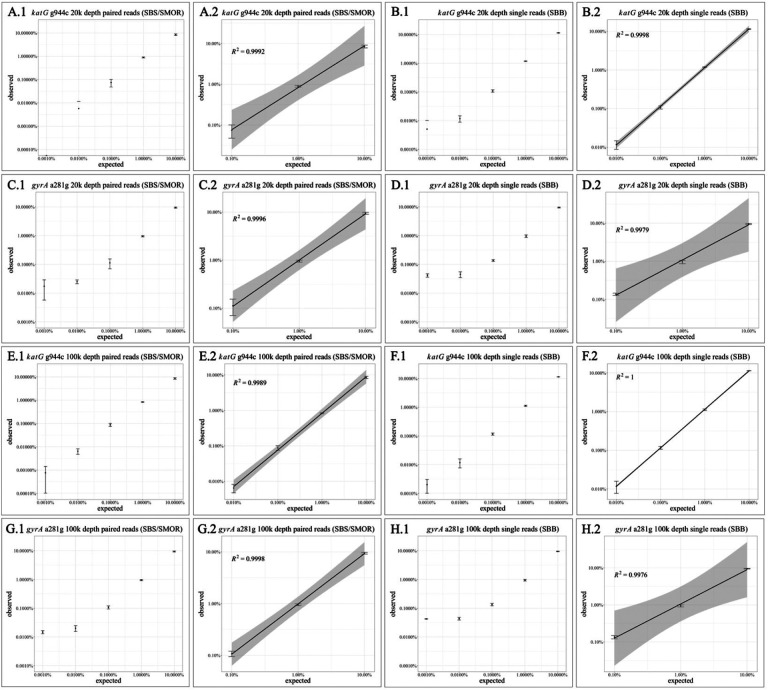
Full range plots for expected versus observed mutations and their associated linear regressions. Vertical bars represent standard deviation from the mean and gray shading on linear regressions indicate 95% confidence intervals. Some vertical bars were missing due to capturing zero sequencing reads for some of the replicates and this occurred only within the very rare mutations *(i.e.,* 0.001% and 0.01%). Plots A.1-D.1 show full range and A.2-D.2 show linear regression results for 20,000x depth for SBS/SMOR and SBB; and plots E-H show same results for 100,000x depth, for targets *katG* g944c (A-B, E-F) and *gyrA* a241g (C-D, G-H).

Targeted deep sequencing analysis: Fastq.gz files generated from the contrived population mixtures were analyzed using the Amplicon Sequencing Analysis Pipeline (ASAP) software [18, 19]. A customized JavaScript Object Notation (JSON) file was created for these *katG* and *gyrA* nucleotide locations [20] and the reads were trimmed of any adapter with any less than 80 nt being removed by bbduk [21]. The trimmed reads were aligned to the target amplicon references using Bowtie2 [22] and the resultant BAM files were analyzed for single nucleotide polymorphisms (SNPs) following the specifications in the JSON file and user defined thresholds including SMOR analysis, if applied (ASAP commands are listed in the supplemental information [20]). ASAP outputs an XML file containing all of the results, which were converted to HyperText Markup Language (HTML) format using a customized extensible stylesheet language transformations (XSLT) output transform [20]. Data from each HTML file was copied, pasted, and manipulated in MS Excel to create the mutation frequencies reported in Tables S1-S4 [20] and the means and standard deviations used to generate the R plots in Fig. 1.

Full and linear range plots: RStudio version 1.3.1056 was used to create full range and linear range plots using ggplot with geom_point for the mean, geom_errorbar for standard deviation vertical bars, geom_smooth for fitting the linear model, and gray shading for 95% confidence intervals of the mean, which used the predict() method. The R code and input files are reported in the supplemental information [20].

Measuring false mutations along sequencing reads: ASAP software was used to find all mutations reported along sequence reads using Illumina read 1 only, Illumina read 2 only, SMOR error correction (overlapping Illumina reads 1 and 2), and PacBio SBB single reads. A customized JSON file was used in tandem with specific user defined thresholds and a customized XSLT output transform file to count all mutations [20]. Data from the HTML output file was copied and pasted into a tab-delimited text file, which was used for further processing. Additional python scripts reported in the supplemental files [20] were used to determine which of these mutations were above the five thresholds (e.g. 10%, 1%, 0.1%, 0.01%, and 0.001%) and create text files to report all mutations. These text files were opened in MS Excel and a countif function was used to determine the exact number of false mutations detected along the sequence reads for each replicate (see the individual counts in the 20k and 100k supplementary files [20]).

Depth plot for the binomial distribution of the five different minor population levels examined: An RStudio installation onto a computing cluster was used to create this plot using parallel computing with 24 cpus and 250GB of memory for each. The R code is reported in the supplemental files [20].

## Results and Discussion

We assess the capabilities of targeted SBB sequencing to identify and quantify low to extremely low minor subpopulations, specifically as compared to error-corrected and non-error-corrected targeted SBS sequencing. Thirty contrived *Mtb* mixtures were created to investigate the performance of these different sequencing and informatics approaches. 10%, 1%, 0.1%. 0.01%, and 0.001% proportions for variants *katG* g944c and *gyrA* a241g were made in triplicate using three different 10-fold dilution series, so each proportion level contained three sample replicates. These variants are linked to resistance to isoniazid *(katG* g944c), a first line drug, and fluoroquinolones *(gyrA* a241g), a second line drug family, and these mixtures were sequenced with SBS and SBB sequencing instruments (see Methods). The SBS sequencing data was analyzed using SMOR error correction (SBS/SMOR) and SBB was analyzed with no error correction.

We first evaluated the expected versus observed mutation proportions at 20,000x read depth, which is a typical depth for previous *Mtb* mutational analyses [8, 11, 13, 15]. The full range is shown for all five mutation proportions in Fig. 1 as well as their associated linear regressions (Fig. 1A–D). SBS/SMOR and SBB gave similar results for minor populations down to 0.1% for *katG* g944c (Fig. 1A and 1B), though SBB demonstrated an extended linear range and a tighter confidence interval for variant detection down to 0.01% (Fig. 1 B.2). For *gyrA* a241g, we observed similar regressions, both with a linear range down to 0.1% (Fig. 1C.2 and 1D.2), however, the 95% confidence interval for the mean was broader for the SBB data. At 20,000x read depth, neither sequencing method detected the 0.001% variant dilution. Lastly, three of the ultra-low data points in these full range plots at 20,000x depth did not have completed standard deviation bars and this was due to capturing zero sequencing reads for some of the replicates in these very rare mutations.

We then evaluated for expected versus observed mutation proportions at 100,000x depth to analyze for ultra-low frequency mixtures (Fig. 1E-1H). This increased depth improved the ability to detect the ultra-low variant frequencies for *katG* g944c down to 0.01% for SBS/SMOR and SBB (Fig. 1E.2, 1F.2, 1G.2, and 1H.2). For *katG* g944c, the 95% confidence interval for SBS/SMOR was wider than for SBB indicating a stronger linear relationship was detected for SBB sequencing from 10%−0.01% (Fig. 1F.2). Alternately, the additional sequencing depth did not improve the ability to detect lower mutation frequencies for *gyrA* a241g with SBS/SMOR or SBB (Fig. 1G.2 and 1H.2).

The sampling depth required to detect the lowest population proportion (i.e., 0.001%) at > 95% confidence is 376,435x or greater for all zero results *(i.e.,* 0/376,435 reads) based on the binomial distribution (see Fig. S1). An accurate zero result at 100,000X *(i.e.,* 0/100,000 reads) with an expected minor population of 0.001% cannot be achieved (p = 0.63). Variants at the 0.001% frequency were detected (Fig. 1E.1), however, these results lack statistical significance at > 95% confidence, presumably due to the lack of sequencing depth.

To assess the false-positive rate *(i.e.,* sequencing errors) at these very low frequencies we searched across the amplicon sequence. A false-positive was defined as a deviation from the plasmid sequence from which the PCR products were derived. This may be an overestimate due to potential PCR error in library prep as well as limitations in detecting variants within the plasmid samples, however, these limitations should apply to both sets of data, enabling a fair comparison. A substantial variation was seen in the total number of variants detected by each technology *(i.e.,* SBS reads demonstrably accumulated more variants than SBB reads) (Fig. S2). Specifically, we analyzed the 171 nucleotide positions along reads for *katG* and the 160 nucleotide positions for *gyrA* where both the SBS and SBB reads overlapped and report the number of false positives using five thresholds *(i.e.,* 10%, 1%, 0.1%. 0.01%, and 0.001%). No false positives were detected for SBS/SMOR or SBB at the 10% and 1% threshold levels for both subsampled depths (20,000x and 100,000x), while SBS without SMOR error correction exhibited false positives for both genes *(katG* and *gyrA)* at the 1% threshold (Table 1). Our results for SBS without SMOR correction are consistent with past observations suggesting minor population detection without any error corrections is limited to between 1–10% for SBS sequencing [10, 16]. In addition, SBS read 1-only exhibited more false-positives than SBS read 2-only; this was likely due to the respective regions reviewed as they were closer to the end of read 1 than for read 2 (Fig. S2). As expected, false As expected, when comparing results for the subsampled 20,000x and 100,000x SBB reads to traditional SBS reads followed by SMOR analysis, a higher percentage of sequenced reads were discarded during SMOR error correction (Tables S1-S4). With a reduction in sequencing depth, the ability for SBS/SMOR analysis to detect minor populations may also be reduced. To remain competitive with the SBB chemistry, 12–18% additional paired reads may be required for the SBS/SMOR method to obtain matching depths. It is also important to note more SBB reads were discarded for *gyrA* than *katG,* which further suggests SBB sequencing exhibited more difficulty sequencing the *gyrA* amplicon in this study.

## Conclusions

Detecting rare genetic variants *(i.e.,* < 1% minor subpopulation) in next generation sequencing data is a critical need for applications ranging from identifying antibiotic micro-heteroresistance in bacteria to detecting low-variant genetic mutations in oncology. As such, a large number of error correction tools have been developed for traditional SBS sequencing to improve its confidence for rare variant detection given its inherent sequencing error. Our results with contrived mixtures for an *Mtb* model system to characterize ultra-low genetic variants demonstrated SBB sequencing chemistry achieved accurate SNP detection down to 0.01% at 100,000x depth and 0.1% at 20,000x depth, without any error correction tools. Traditional SBS sequencing is unable to achieve this accuracy without the use of sophisticated error correction tools *(e.g.,* SMOR, UMI, duplex and others). One limitation of SBS/SMOR analysis is that it is designed specifically for targeted genomic regions from PCR products. In contrast, SBB sequencing looks very promising for both targeted and unbiased whole genome sequencing, as its innate accuracy can be used on non-amplicon reads to detect minor subpopulations with confidence to less than 1% of the total population. The utility of SBB chemistry is that it reduces sequencing depth requirements, removes potentially complicated bioinformatic downstream pipelines, and allows for accuracy that rivals the best performing tools developed to enhance traditional SBS sequencing.

## Figures and Tables

**Table 1.** Sequencing errors* in *katG* and *gyrA* sequence reads for SBS versus SBB

A.	SBS read 1 *kalG*	SBS read 2 *kalG*	SBS/SMOR *kalG*	SBB *kalG*	SBS read 1 *gyrA*	SBS read 2 *gyrA*	SBS/SMOR *zyrA*	SBB *gyrA*
Threshold	20,000x	20,000x	20,000x	20,000x	20,000x	20,000x	20,000x	20,000x
10.000%	1±0	0	0	0	0	0	0	0
1.000%	39±3	1±0	0	0	14±1	2±0	0	0
0.100%	216±9	30±4	7±2	9±3	134±3	32±5	4±1	12±7
0.010%	539±7	346±8	213±6	171±8	401±14	322±17	196±7	175±9
0.001%	678±11	463±6	337±3	263±12	528±8	414±5	319±4	268±10
B,	SBS read 1 *kalG*	SBS read 2 *kalG*	SBS/SMOR *kalG*	SBB *kalG*	SBS read 1 *gyrA*	SBS read 2 *gyrA*	SBS/SMOR *gyrA*	SBB *gyrA*
Threshold	100,000x	100,000x	100,000x	100,000x	100,000x	100,000x	100,000x	100,000x
10.000%	1±0	0	0	0	0	0	0	0
1.000%	38±1	1±1	0	0	15±1	2±0	0	0
0.100%	214±5	28±0	5±1	10±2	132±2	31±5	3±1	11±10
0.010%	520±6	324±10	219±8	149±10	398±3	299±8	191±2	151±4
0.001%	995±9	601±1	504±7	422±7	777±31	556±5	490±1	411±10

* Number of false positives reported along *katG* and *gyrA* sequence reads for SBS read 1, read 2, or both overlapping reads (SMOR); and for SBB single reads at A) 20,000x depth and B) 100,000x depth.

## Data Availability

Supplemental materials were uploaded to the Zenodo Repository.
